# Penis deformity after intra-urethral liquid paraffin administration in a young male: a case report

**DOI:** 10.1186/1757-1626-1-223

**Published:** 2008-10-07

**Authors:** Ioannis Kokkonouzis, Georgios Antoniou, Athanasios Droulias

**Affiliations:** 1 Accident and Emergency Department, Kyparissia General Hospital, Kyparissia, Greece; 2 Department of Urology, Kyparissia General Hospital, Kyparissia, Greece

## Abstract

**Background:**

Self-induced injections of liquid substances mainly for penis enlargement is a well-documented but still rather uncommon practice in the western world.

**Case presentation:**

Herein we present the case of a 30-year-old male who self-inflicted, twice in a six-month-period, intra-urethral liquid paraffin and tied up his penis with a cord in order to achieve both enlargement and elongation. He arrived in our emergency department suffering from suprapubic pain; physical examination revealed a rather unique deformity of the penis. He finally refused any treatment and absconded.

**Conclusion:**

Side effects after paraffin administration are various and sometimes severe. Most of the times surgical treatment is needed and radical excision together with follow-up seems the best modality. Such practices emphasize on the public's misbelieves and that some aspects of sexual medicine are yet covered with the veil of misconception.

## Background

Injections of mineral oils, like paraffin, have been used, more often in Eastern Europe and Asia, subcutaneously and according to our report, even intra-urethral, intending mainly to penile augmentation. Such materials can not be metabolized and a foreign-body-reaction occurs. As a result, health risks may need immediate intervention. This case also emphasizes on the inability of modern medicine to uncover established public misconceptions.

## Case report

A 30-year-old heterosexual male immigrant of Bulgarian origin presented to the emergency department complaining of suprapubic pain which started two days prior to admittance. No history of trauma, TB infection or previous surgery was reported whatsoever. He stated normal sex life, normal erection and ejaculation during intercourse. At the time of observation his vital signs were in normal range and on physical examination he presented suprapubic tenderness and his penis was noted to be abnormally large in circumference, painless but stiff to the feel with a markedly stricture at the middle of the penis shaft. The skin was depigmented in many areas largely at the base. No enlargement of the inguinal lymph nodes was noted (Legend 1). On digital examination a tender and mildly painful prostate was the main finding.

**Figure 1:**
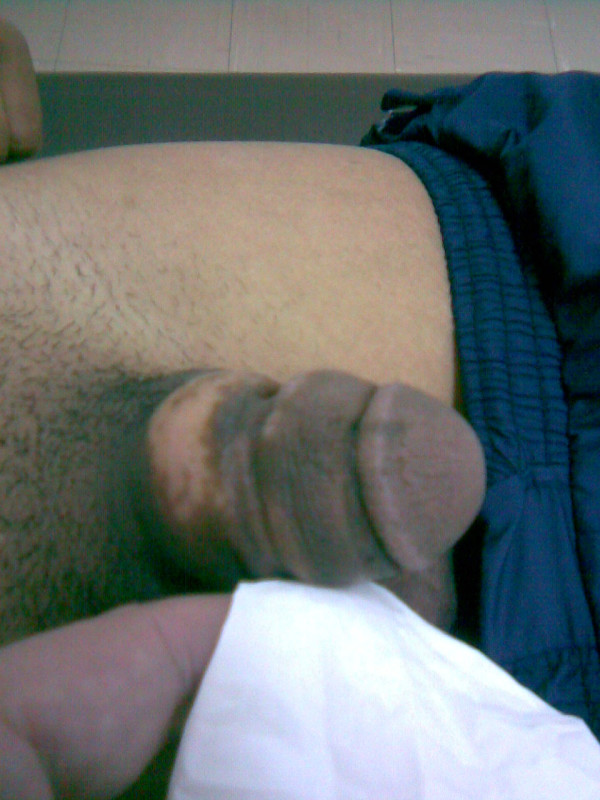
**A rather unique deformity at the penis of this young male has been the result of intra-urethral infusion of paraffin as this figure demonstrates**. Note the examiner's finger.

At that point the patient reluctantly confessed that he has infused approximately 8–10 milliliters of liquid paraffin into his urethra by a small syringe some six months before and repeated the infusion three months ago. His purpose was to achieve penis enlargement. In order to avoid the entrance of the substance inside the rest of the urinary tract he tied a string around the base of the penis during the time of the infusion (matching to the decolorized region of figure 1). The routine laboratory tests were unremarkable as well as the culture of urine specimen, which was sterile, while total PSA was slightly increased. During his short stay at the emergency department a suprapubic ultrasonographic evaluation was performed revealing moderate increase in prostatic diameter, borderline obfuscation and lateral lobe microcalcifications. Our patient refused any further urological consultation or psychiatric evaluation, strongly denied any treatment and absconded.

## Discussion

Some cases of subcutaneous injected liquid substances like paraffin or vaseline into the urogenital region can be found in the literature mainly on purpose of penis circumference enlargement. This pathological entity is known as penile paraffinoma. [1-8] In case no pathological finding is revealed, physical examination together with medical history, with emphasis on paraffin injections into urogenitalia, can presumably establish the diagnosis. It's not unusual that the formation of the granuloma appears even years after the injections. Eandi et al. in a case of a 71-year-old man reported that the development of the paraffinoma occurred forty years after a series of penile injections of unknown substances.[8] Moon et al. contacted a survey on 357 imprisoned men who had injected mineral oils into the urogenital region with the use of a semi-structured questionnaire combined with psychological evaluation. At the majority of the respondents (78%) non-medical personnel has performed this procedure and their main motive was the fear of inferiority of their penis or the weak erectile function. Following the injection two thirds found no relief, nearly everyone (91%) was not satisfied with his penis afterwards and finally more than two-thirds wanted to remove the injected material. [9] More recently, Pehlivanov et al. in a twenty-five-patient cohort study reported that the motivation of the majority of them was to enlarge penis's size and secondly to increase their sex partners' intercourse satisfaction. This study also emphasizes on the fact that non-medical personnel (60%) or the patients themselves (40%) did the injections. As the study was retrospectively contacted in a dermatology clinic it is really interesting that all of them had awkward social behavior (prisoners and beggars).[10]

Herein we report a case of self-induced intra-urethral paraffin infusion for penis elongation and circumference enlargement in a young heterosexual male who was working as a farm worker. The route of administration is extremely unusual and it seems that the lyophilic nature of the substance deceived our patient into believing that it could diffuse the material into the corpora cavernosa thus inducing their hypertrophy. Our patient stated that he did not used needle during the procedure so it seems reasonable enough to conclude that the effusions were made after he tied up his penis with the cord. At the same time a second cord was tied around the penis shaft proximally. Whilst the most likely diagnosis for this patient, at the time of arrival at the emergency department, was acute prostatitis, we found it difficult to prove a causal relation between the current state of disease and the repeated intra-urethral infusions of liquid paraffin. In such cases a diagnostic dilemma is present given the observed latency period, after which an approved relationship can not be established.

Side effects could be disastrous, occur in a relatively short period after and concern the vast majority of patients. According to the Moon et al. study only 15, 6% did not experience any abnormalities.[9] At their review study including 26 cases of sclerosing granuloma, Lee et al. reported that the symptoms' onset time was approximately 18, 5 months.[11] Pehlivanov et al. reported that the period between the infusion and the onset of complications was about one year, when 56% of the injected patients revealed severe side effect (fistulas and wide ulcers).[10] So urethral stenosis, inflammation, repeated urinary track infections, erectile dysfunction of variable severity, severe ulcers and fistulization seem to be the most common side effects one has to come up with due to injected liquid substances. [9-11] The potential co-appearance of penis squamous cell carcinoma, especially in elderly patients when ulcerating lesions are present, must always be taken into account and furthermore needs urgent handling. [9-12] Moreover psychological evaluation is needed to clarify any underling psychiatric disease although Moon et al. found no evidence of psychiatric pathology whatsoever at their cohort-study. [9]

All patients must be encouraged to receive treatment and radical surgical excision has been proposed as the most appropriate method to avoid future symptoms. This procedure includes total excision of the foreign body granuloma combined with repair surgical techniques with the use of scrotal flaps or split thickening skin grafts when this is necesssary.[10,11,13] Lee et al. in a 26-case review study reported that after complete excision and appropriate penoplasty everyone but two of the patients had favorable outcome.[11] The same result has been reported in every single case in which patients received surgical treatment in both Pehlivanov et al. and in Jeong et al. studies, the last one including 17 patients, without any complaints or erectile disturbance during follow-up.[10,13] As a result close collaboration of urologists and plastic surgeons and a well-scheduled follow-up is needed in order to avoid surgical complications or future recurrences.

On the other hand there are some reports suggesting a rather more conservative approach in selective patients. Rosenberg et al. proposed in a three-patient study that more conservative measures like minimal surgical interventions and short-term use of antibiotics should be considered especially when patient 's cooperation is laking.[14] Akkus et al. in a 42-year-old man, suffering from an irregular penile mass with multiple ulcers, voiding difficulty and erectile dysfunction due to repeated vaseline injections, performed local treatment with intralesional triamcilone and hot-water baths as the patient refused any surgical intervention.[6] Lawrentschuk et al. also treated one patient with oral corticosteroids for a six-week period resulting in the disappearance of the granuloma. The authors suggested that surgery should be reserved for recurrent or refractory cases after the failure of steroid treatment. Biopsy in order to exclude malignancy is therefore needed when no surgical treatment would be performed.[15] Unfortunately our patient strongly refused any intervention.

## Conclusion

In conclusion we presented a case of a young male who repeatedly self-infused, intra-urethral liquid paraffin to achieve penis enlargement. Such cases reflect not only the insufficiency in public health information, especially in the developing world, but also the inability of modern medicine to prove its reason and dissolve popular misconceptions.

## Authors' contributions

All of the authors were involved in the management of the case. IK and GA were involved in the process of writing the manuscript and AD did the final corrections. All authors have read and approved the final manuscript.

## Consent

Written informed consent was obtained from the patient for publication of this case report. A copy of the written consent is available for review by the Editor-in-Chief of this journal.
